# Efficacy of disinfectant-impregnated wipes used for surface disinfection in hospitals: a review

**DOI:** 10.1186/s13756-019-0595-2

**Published:** 2019-08-19

**Authors:** Xinyu Song, Lutz Vossebein, Andrea Zille

**Affiliations:** 1 2C2T – Centro de Ciência e Tecnologia Têxtil, Universidade do Minho, Campus de Azurém, 4800-058 Guimarães, Portugal; 2 Faculty of Textile and Clothing Technology, Niederrhein University of Applied Sciences, Webschulstrasse 31, 41065 Mönchengladbach, Germany

**Keywords:** Disinfectant-impregnated wipe, Ready-to-use wipe, Surface disinfection, Interaction, Efficacy, Infection control

## Abstract

**Background:**

“Ready-to-use” disinfecting wipes (also known as pre-impregnated disinfecting wipe) are broadly used in food industry and domestic situations. Their application in hospitals and healthcare centres for decontamination of medical devices and surfaces is steadily increasing because of their convenient implementation in practice and reliable performance. Beside their acceptable compliance and easy application, literature reported the disinfection failure due to the interaction between textile substrate and active ingredients, which can highly increase the risk of an infection outbreak. This review aims to call attention to the wide range of variables affecting the disinfectant-impregnated wipes’ (DIWs) disinfection performances in hospitals.

**Methods:**

A systematic literature search based on the five categories i. wipes, ii. disinfectants, iii. Application methods, iv. interaction between wipes and active ingredients and v. wiping strategy which can possibly influence the disinfection effectiveness of DIWs was conducted by Google scholar. Studies regarding the efficacy evaluation of DIWs in clinical applications were also reviewed from the National Centre for Biotechnology Information database.

**Results:**

Variables that impact on the disinfection performance of disinfectant-impregnated wipes in surface disinfection in hospitals were summarised and critically discussed. In addition to the information, current disinfectant-impregnated wipes’ decontamination efficacy test standards were reviewed, and different standards exposed some disadvantage in their testing design.

**Conclusion:**

Various parameters contribute to the impact of DIWs disinfection performance in practice. The interaction between disinfectant active ingredients and the wiping materials barricades their broad application in hospitals. More studies of the DIWs’ disinfection efficacy in clinical practice are in need. Current standards evaluating the DIWs’ efficacy are required to improve for more realistic condition simulation and differentiating between mechanical removal of inoculum from a surface and chemical inactivation of the test microbe.

## Background

Healthcare-associated infections (HCAIs) caused by the transfer of nosocomial pathogens from high-touch environmental surfaces and medical devices are responsible for significant patient morbidity, mortality and economic cost [1–5]. More recently, evidence shows nosocomial pathogens, including methicillin-resistant *Staphylococcus aureus* (MRSA), norovirus, *Clostridium difficile*, vancomycin-resistant *Enterococcus*, and *Acinetobacter* species etc. shed by patients can contaminate hospital surfaces at concentrations sufficient for transmission, surviving for extended periods and persisting despite attempts to remove them [5–11].

An effective cleaning and disinfection practice, such as chemical disinfection, heat, and ultraviolet germicidal irradiation etc. play a key role in preventing cross-contamination [12, 13] and spread of HCAIs [14–19]. Among all the surface disinfection approaches, the utilization of chemical disinfectant is broadly diffused in food industry, hospitals and healthcare centres because of its easy application and broad spectrum of microbicidal activity [20, 21]. In the application of disinfectant in practice, the “ready-to-use” disinfecting wipes (RTUDW) (also reported as pre-impregnated disinfecting wipes, pre-saturated towelette and pre-wetted disinfecting wipe in some literatures) are increasingly accepted for decontamination of high-touch surfaces because of their convenient implementation and reliable performance [22–24].

Although disinfecting wipes have been widely used and spread in hospitals for decontamination of medical devices or high-touch environmental surfaces [25], the effectiveness of their disinfection performance is always in discussion.

Disinfectant-impregnated wipes (DIWs) basically consists of towels saturated with diluted disinfectant as well as other chemical products such as surfactants, preservatives, enzymes, and perfumes etc. When two materials encounter, the interaction between each other is not negligible and often has an influence on their original function. The factors that could possibly influence the system’s disinfection efficacy focus on the following aspects: wipe, disinfectant, application method, the interaction between each other as well as the wiping strategy and storage time. Going through the studies regarding the efficacy of DIWs in clinical practice, the authors believe that more attention needs to be addressed in this topic. The review also points out the need for improvement for disinfecting wipe decontamination efficacy testing standards. Since it is an important validation step before the disinfectant-impregnated wipe products launched into the market and further used in the hospital.

## Methods

Several parameters influencing the antimicrobial efficacy embracing the external factors for instance target surface (material, organic load), target microorganism, ambient environment (temperature, humidity), and internal factors such as the disinfectant (type, concentration), wipe (material type, construction/fibre architecture), application method, wiping strategy, are investigated by numerous researchers. A systematic literature search focusing on the internal factors were conducted by Google scholar. General efficacy study of DIWs in clinical practice was searched on NCBI (National Center for Biotechnology Information) database. For additional information related to the efficacy testing protocols, standards were explored and reviewed under the scope of EU standards issued by the European Committee for Normalization (CEN), Technical Committee 216 (TC 216) and US standards by the United States Environmental Protection Agency (EPA) Office of Pesticide Programs (OPP) in cooperation with AOAC International and ASTM International. In addition, guidelines i.e. Guidance on the Biocidal Products Regulation from European Chemicals Agency (ECHA) and Guideline for Disinfection and Sterilization in Healthcare Facilities by Healthcare Infection Control Practices Advisory Committee (HICPAC) of Centres for Disease Control and Prevention of USA (CDC) are also included for the review.

Data were extracted by two authors based on the examination of the titles and abstracts obtained from Google Scholar and PubMed database. Meanwhile, one author reviewed the abstracts to check if the material fulfils the criteria of the work. Subsequently, full articles deemed necessary for the review were obtained and reviewed by another author.

## Results and discussion

The disinfection process of a disinfectant-impregnated wipe (DIW) can be divided into two parts that constitute the overall decontamination activity. One part is related to the microorganisms taken away by the wipe itself by means of mechanical action. The other part is related to the active microbicidal action of the disinfectant solution released by the wipe on the surface. The parameters that influence its efficacy, as well as the effectiveness studies of DIWs in literature are exhibited as follows.

### Wipe

The wipe for disinfection is mostly made of textile materials, including, but are not limited to, cellulosic fibres (cotton, woodpulp, viscose, lyocell) and thermoplastic fibres (polyethylene terephthalate, and polypropylene). Particularly for disposable wipes, the raw materials are normally inexpensive like cellulosic fibres and polyolefin fibres. Cellulosic fibres are used to ensure high water retention and storage capacities and polyolefin fibres are accountable for high tensile strength, abrasion and solvent resistance [26]. The majority of wipes for surface disinfection in the market are made of blends of polyester and viscose fibres/woodpulp [26, 27].

Wipes offer a cleaning procedure by the mechanical action of wiping, which can remove the organic debris along with the disinfection activity.

Likewise, the microorganism can be mechanically removed by the wipe. However, attention should be paid to the transfer of microorganism to other parts of the surface. The removal of the microorganism depends on the inherent properties of the wiping material such as surface energy, fabric structure and fibre types as well as by the applied pressure force, the geometry of the mechanical action, the number of passages and type of microorganism adhesion mechanism [28, 29]. As stated before, it is also important to consider that during the wiping action some microorganisms could be just transferred in another place of the treated surface instead of being removed. This transfer depends by the wipe retaining ability and by the bactericidal activity of the disinfectant adsorbed into the wipe [28].

The disinfectant solution released by the wipe on the target surface is mainly responsible for the bactericidal activity. The quantity and concentration of active ingredient and the amount of the solution remaining on the surface are important efficacy indicators and depending on the interaction between the wipe and disinfectant. Also, the amount of released solution is highly dependent on the wipe absorbent property. Without any doubt, wipe plays an important role in decontamination of the target surface. Table 1 below looks at available advance wipes.

**Table 1:** Advanced wipes in the market and their advantages and disadvantages. [30–35]

Advanced wipes	Description	Advantages	Disadvantages	Ref.
Microfiber wipes	Microfiber wipe relates to wipe made from fibres whose diameter is in the range of micro scale.	Its cleaning and disinfection efficiency have been evaluated by numerous studies. Some demonstrated that microfiber system has superior microbial removal efficiency compared with cotton string mops.	Others stated that the use of microfiber cloth spread the bacteria although there was an overall reduction in bacterial counts on the contaminated surface.	[30–33]
Composite wipes	Composite nonwovens wipes are composed of a mixture of fibres and particulates or of fibres that differ in their chemistry, denier or shape in order to provide improved functionality at lower cost.	The advantage of composite wipes is their good durability maintaining at the same time good absorbency properties.	Different materials composition may limit the production process choice.	[30–33]
Biodegradable wipes	The nonwoven fabrics are usually composed of cotton fibres thermal bonded using bio-based thermoplastic polymers.	Providing the soft and absorbent property from cotton alongside the increased strength by the synthetic biodegradable fibres. Biodegradable wipes are of great interest for their obvious environmental and sustainability advantages.	More cost in the aspect of material	[34, 35]
Flushable wipes	Flushable nonwoven wipes are designed to be able to be flushed down the wastewater system without adversely impacting plumbing or wastewater infrastructure and operations.	A relief to landfill management as waste in the concern of environment protection and sustainable development. Flushable nonwoven is strongly supported by the industry.	There is technical difficulty with flushable wipes: the wipe must break down immediately in a toilet bowl and be small enough to be transported from the toilet bowl to the sewage system in a single flush without causing clogging, blockages or equipment failure in the wastewater conveyance and treatment systems but at the same time it has to maintain strong enough to be stored and used when wet.	[34]

### Disinfectant

Disinfectant as the main constituent for disinfection action has a crucial impact on the decontamination process. The antimicrobial activity of disinfectants performs in two different ways: growth inhibition (e.g. bacteriostatic, fungistatic) and lethal action (sporicidal, bactericidal, fungicidal, and virucidal effects) [36]. Disinfectants comprise a wide variety of active chemical agents (biocides). The active ingredients found in the market are generally alcohols, chlorine, aldehydes, peroxygens, and quaternary ammonium compounds [37]. Every type of disinfectant presents some advantages and disadvantages allowing, or not, its use in wipes.

Alcohol is cheap and easy to obtain allowing an efficient wetting of the surfaces with a rapid bactericidal effect without bacteriostatic action and relevant toxicity issues. However, it is highly inflammable, corrosive to metals with lack of efficacy in the presence of organic debris and tends to swell and harden rubber and certain types of plastics. It is not sporicidal and has low effectiveness in the inactivation of some type of virus. Moreover, due to its high volatility, it is difficult to ensure enough contact time in open systems [38–42].

Under the chemical family of chlorine compounds, the most used disinfectants are hypochlorite, chlorine dioxide and the chloramine-t trihydrate. Hypochlorite is the most used chlorine disinfectants especially because of its low cost and fast mode of action. It displays a large bactericidal spectrum with no toxic residues, and it is not affected by water hardness. However, it is also corrosive to metals (> 500 ppm), easily inactivated by organic matter, irritating and burning for skin, eyes and mucous membranes. It can discolour and bleach textiles and can become very dangerous in contact with ammonia or acid due to the generation of toxic chlorine gas. Chlorine dioxide also shows a wide spectrum of biocidal activity including mycobacteria with short contacts time. It provides prolonged bactericidal effect than chlorine due to its high retain of antimicrobial active ingredients but with long-term use can damage the outer plastic coat of some insertion tubes. Chloramine-t trihydrate it is able to retain chlorine for a long time which results in a more prolonged bactericidal effect, however, occupational asthma has been reported due to its prolonged exposition [25, 43–45].

Hydrogen peroxide presents a satisfying germicidal activity including bacterial spores (with longer contact time). It is relatively environmentally friendly due to its fast degradation. An accelerated hydrogen peroxide (AHP) was specifically developed for widened material compatibility and application variability. It can cause chemical irritation resembling pseudomembranous colitis. Peracetic acid (PAA) has a rapid action against all microorganisms (inclusive spores at low temperatures) at low concentration. It is very efficient even in the presence of organic matter, without producing residues. However, it is unstable, particularly when diluted, and is corrosive to copper, brass, bronze, plain steel, and galvanized iron (corrosion decline by additives and pH modifications) [46–51].

Quaternary Ammonium Compounds (QACs) are the most commonly used disinfectant in ordinary environmental surfaces with a good cleaning and deodorization property and most important a broad spectrum of biocidal and sporostatic activity (lipid, enveloped viruses). Incorporation of quaternary ammonium moieties into polymers showed an effective antimicrobial effect against biofilm. Nowadays, QACs are the most used disinfectant in wipes. However, they also have some drawbacks such as a susceptibility to high water hardness and low efficacy against gram-negative bacteria and non-enveloped viruses. Moreover, numerous studies showed that the adsorption of QACs onto the cotton substrate wiping material could lead to the failure of the disinfection process [47, 52–58].

Summarise the information, the preferred disinfectants used for disinfectant -impregnated wipes in the market are quats-alcohol wipes, hydrogen peroxide wipes, hypochlorite wipes due to their user-friendly feature. Emphasis is drawn to the safety issue for the staff during their application. These DIWs are extensively approved to be employed in hospitals and healthcare centres. More information regarding the disinfectant and their application evaluation can be found in Table 2.

**Table 2:** Active ingredients, chemical formulas, pros and cons of disinfectant-impregnated wipes applications

Disinfectant category	Example of active ingredients	Chemical formula	Advantages	Shortcomings	Ref.
Alcohol	Ethyl alcohol (Ethanol)	C_2_H_6_O	Rapid bactericidal effect. No bacteriostatic action. Relatively cheap and easy to obtain. Wet the surface easily.	Tend to swell and harden rubber and certain plastics. Not sporicidal. Inflammable. Poor inactivation effectiveness was reported for some virus. Lack of efficacy in the presence of organic debris. Metal corrosive. Difficult in ensuring certain contact time in an open system.	[38–42]
	Isopropyl alcohol (Isopropanol)	C_3_H_8_O			
Chlorine and chlorine compounds	Hypochlorites	ClO^−^	Most used chlorine disinfectants. Large bactericidal spectrum. No toxic residues. Not affected by water hardness. Inexpensive and fast mode of action.	Corrosive to metals (> 500 ppm). Inactivated by organic matter. Irritating and burning for skin, eyes and mucous membranes. Discolour and bleach textiles. Toxic chlorine gas formation in contact with ammonia or acid.	[25, 43–45]
	Chlorine dioxide	ClO_2_	Wide spectrum of biocidal activity. Efficient mycobactericidal activity in short contacts time. It provides prolonged bactericidal effect than chlorine due to its high retain of antimicrobial active ingredients.	Long-term use can damage the outer plastic coat of some insertion tubes.	
	Chloramine-t trihydrate	C_7_H_7_ClNNaO_2_S	Chlorine retains longer which results in more prolonged bactericidal effect	Occupational asthma has been reported.	
Peroxygens	Hydrogen peroxide	H_2_O_2_	Satisfying germicidal activity including bacterial spores (with longer contact time). Environment friendly due to its fast degradation. Accelerated hydrogen peroxide (AHP) was developed with widened material compatibility and application variability.	May have chemical irritation resembling pseudomembranous colitis	[51]
	Peracetic acid (PAA)	C_2_H_4_O_3_	Rapid action against all microorganisms at low concentration. Reinforced removal of organic material without residue. Effective in the presence of organic matter. Sporicidal at low temperatures	Corrosive to copper, brass, bronze, plain steel, and galvanized iron. (corrosion decline by additives and pH modifications) Unstable, particularly when diluted.	
Quaternary ammonium compounds (quats or QACs)	Alkyl dimethyl benzyl ammonium chloride	C_22_H_40_N^+^	The most commonly used disinfectant in ordinary environmental surfaces with broad spectra of biocidal activity (lipid, enveloped viruses). Sporostatic. Good cleaning and deodorization property. Incorporation of QA moieties into polymers presents effective antimicrobial effect against biofilm.	Numerous studies show the adsorption of QACs onto the cotton substrate wiping material, which could lead to the failure of disinfection process. Susceptible with high water hardness. Less effective with gram-negative bacteria and non-enveloped viruses.	[47, 52–58]
	Benzyl dimethyl octyl ammonium Chloride	C_17_H_30_ClN			
	didecyl dimethyl ammonium chloride	C_22_H_48_ClN			

### Application method

When applying the surface disinfectant on the target surface, the approaches can be generally divided into two groups: i) without mechanical action, e.g. total immersion and directly spraying, and ii) with mechanical action, e.g. spray & wiping, dipping & wiping, and soaking & wiping [59]. The main benefit from the mechanical action is its ability to remove the organic debris that could hinder the disinfection action and DIWs falls in the category with mechanical action.

The “Spray and Wipe” method starts with a direct spray of the disinfectant solution with an aerosol or trigger sprayer on the target surface, followed by a wipe of the target surface. The spray action allows direct contact of the disinfectant solution with the target surface. However, there are several drawbacks such as possible overspray, difficulty in covering surfaces (undersides of bedrails), and generation of atomized disinfectant in the air that can subsequently be breathed by workers and patients [60]. Due to the flammability of numerous sprayed disinfectants, the presence of open fires during use have to be taken into account [61].

“Dip and Wipe” means dipping a dry towelette into one disinfectant solution for 5–10 s, wring out the excess solution and directly use it for disinfecting hard surfaces. The short contact time that the wipe spends in the disinfectant solution can limit the concentration of active ingredients applied on the target surface. A towelette carrying an insufficient amount of surface disinfectant may lose its antimicrobial activity and later becomes itself a potential vehicle of pathogen transmission [62]. In addition, the inappropriate reuse of the towelette may promote the accumulation of microorganisms and raise the risk of cross-contamination during the disinfection process [63–65].

“Soak and Wipe” method, also known as “bucket method”, was widely used for disinfection processes in hospitals. Similar to the “Dip and Wipe” method [66, 67], the towelette is soaked into disinfectant solution from 10 min up to 8 h instead. Before use wring out the excess solution and directly applied to a hard surface. The “Soak and Wipe” method was the most prevailing methods among all, owing to its acceptable performance and easy application. It allows a relatively long contact time ensuring enough active ingredient load in the towelette before use. Nevertheless, there are some studies reporting possible interactions between wipes and disinfectant due to the longer soaking time resulting in reduced antimicrobial activity of disinfectants [52, 68, 69]. Moreover, a chemical binding of the disinfectant to the wipe could lead to a decrease of disinfectant concentration in the bulk solution [69]. As indicated in the aforementioned method, improper reuse of the towelette can result in cross-transmission of pathogens on the treated surfaces [63–65].

Ready-to-use disinfecting wipe (Abbreviated as RTUDW) is a pre-wetted towelette containing disinfectants, antiseptics, surfactants etc. in a sealed package ready for use in surface disinfection up to 1 month (shelf life can be longer). This method is also known as “pop up” wipe in hospitals. The use of RTUDW is steady increasing partially profits from the rapid development in nonwoven technologies, which provides a relatively good cost performance [70]. The RTUDW is designed to be used without any preparation time. Considering the compliance, employee time, and costs, RTUDW is highly recommended for the surface disinfection [71]. It has been tested in many research projects to be proved to possess good antimicrobial effect in several conditions [40, 64, 72]. RTUDW is disposable, which eliminates the possible contaminations and transfer of pathogen due to towelettes reuse [64]. However, the longer storage time could increase the probability of losing antimicrobial activity due to the possible binding of active ingredients onto the towelettes or by the degradation of the active ingredient [73]. Some research shows RTUDW’s bactericidal efficacy decreases on wiped larger surface areas [74]. In addition, the disposable property could be a problem with waste management.

All above-mentioned application methods can be found in use in practice for different surfaces. “Spray and Wipe” and “Dip and Wipe” are not recommended for surface disinfection in general due to many drawbacks previously listed. “Soak and Wipe” is still commonly used in the hospital for daily cleaning and disinfection of large high-touch environmental surfaces such as floors, tables, lockers, examination couches. However, in this method, a potential interaction between disinfectant solution and wiping material can decrease the efficacy. The most prominent method is ready-to-use disinfecting wipes. Considering the antimicrobial efficacy of commercial wipes is already qualified by required standards before released into the market, there is less possibility of disinfection failure with this method. Nevertheless, ageing of the products needs to be further investigated as well as other parameters (e.g. wiping area, wiping passage, etc.) during the wiping process should be clarified by the manufacturer on the package.

### Interaction between wiping material and disinfectant

A few investigations have been performed evaluating the interaction between wiping material and surface disinfectant. Unfortunately, nearly all of them were exclusively focusing on the interaction between quaternary ammonium salts (quats) and cotton substrate. Bloss et al. (2010) have classified the absorption of active ingredients onto textile substrate by testing three different surface disinfectants and four different types of fabrics. They found out that the exposure of diluted surface disinfectants to various types of fabrics resulted in considerable adsorption of active ingredients [69]. Additionally, Boyce et al. (2015) found that several factors, including the soaking time and quats binding to specific wiping material, influence the efficacy of quats-based disinfectants. However, their experimental design showed two severe limitations: i) the wipes were taken out for quats concentration test in chronological order and the adsorption of wipes accounting for the decrease of quats concentration in the bucket was not taken into consideration; and ii) the lack of microbiological tests can hardly determine whether the low concentrations of quats released from the three wiping materials resulted in less potent reduction of bacterial counts on surfaces [68]. The investigation of Hinchliffe et al. (2016) may be the first comprehensive study of the possible parameters from both perspectives of textile substrate and disinfectant solution influencing the quats binding degree onto a cotton substrate. They found that the amount of alkyl-dimethyl-benzyl-ammonium chloride (ADBAC) depleted from solution varied with the liquor ratio, pH, temperature, concentration of electrolytes and type of pre-treatment applied to the textile substrate. However, their investigation only measured the adsorption of active ingredients onto textile substrate in the bulk solution instead of the loss of active ingredients during the application stage (resulting from the binding of the active ingredients on the textile substrate). [52, 75]. Later, they demonstrate that quats adsorption onto cotton substrate can be minimized and maintain the efficacy against gram-negative (*Pseudomonas aeruginosa*) and gram-positive (*Staphylococcus aureus*) bacteria. [76]

In summary, disinfectant concentration, material compatibility, contact time, liquor ratio (wipe mass/disinfectant solution volume), an additive of other chemistries, and temperature are possible parameters impacting on the interaction of disinfectant and wipes.

### Wiping strategy

Several studies have shown that high-touched surfaces and devices can serve as a route for transmission of pathogens [77–79]. However, proper disinfection protocols and wiping strategies are still in development.

Wiping strategy includes the applied pressure force, wiped surface area, the geometry of the mechanical action, the number of passages etc. One recent study from A. M. West et al. tested the bactericidal efficacy of ten ready-to-use disinfectants in the form of pre-wetted towelettes [74]. The objective of the study focusses on the impact of surface area(s) wiped on its bactericidal efficacy. The result implicates a larger wiping surface area may lead to decreased bactericidal efficacy. However, rare attention is given for this factor, especially a severe lack of consideration from the efficacy testing standards. Detailed information is given in the next section.

### Standards for disinfecting wipes’ efficacy test

In the last decades, numerous regulations and standards have been issued by various organisations for testing the efficacy of the disinfectants. The standards cover the most important factors that influence the effectiveness of a disinfectant, such as the target microorganism (bactericides, mycobactericides, sporicides/sterilants, fungicides, tuberculocides and virucides), the target surface (tile, stainless steel, wall panels, glass, etc.) and the application strategy (liquid, with wipe or spray method). Many protocols have been designed to validate the disinfectant’s efficacy at the concentration commonly used against a panel of clinically significant microorganisms on the surfaces most routinely disinfected.

In EU, the disinfectant efficacy test is regulated and issued by the European Committee for Normalization (CEN), Technical Committee 216 (TC 216) under the work program “Chemical Disinfectants and Antiseptics” [80]. Two phases were developed for assessing the disinfectant effect: 1) Phase 1 is mainly suspension-based tests for the basic evaluation of disinfectant efficacy against different microorganisms, apart from mycobacteria, under clean conditions. It is applied to evaluate the bactericidal (EN 1040), sporicidal (EN 14347) and fungicidal (EN 1275) activity of chemical antiseptics and disinfectants when appropriate standards are not available. It is a minimum requirement for the assessment of basic biocidal activity under generic conditions (food, industrial, domestic and institutional, medical and veterinary areas). 2) Phase 2 is designed for evaluation of the bactericidal, sporicidal, fungicidal and virucidal, activity of chemical disinfectants applied individually in specific conditions such as food, industrial, domestic, institutional, medical and veterinary areas. In the scope of Phase 2, European Norm is divided into two steps. Step 1 is a suspension test while step 2 is a carrier-based test. Both as suspension-based tests, Phase 2, step 1 test is prior than Phase 1 not only because the application area is more specific in the test but also because it introduces the dirty conditions in testing the performance of surface disinfectant with the involvement of organic debris (Phase 1 only tests clean conditions). The dirty condition is able to demonstrate if a product (surface disinfectant) reacts with other substances such as proteins. Unfortunately, the suspension-based test is far away from the disinfectant performance in real practice. Consequently, carrier-based tests were developed to fulfil the need for disinfectant efficacy evaluation to various surfaces (instruments, surfaces, etc.) under practice-oriented conditions. Notably, in the carrier-based test, there are standards used for non-porous surfaces and porous surfaces with and without mechanical action. In the case of disinfecting-impregnated wipes, it applies to the standards for non-porous surfaces with mechanical action. In conclusion, standard EN 16615 is the most suitable one.

In the US, the United States Environmental Protection Agency (EPA) Office of Pesticide Programs (OPP) has the responsibility for regulating antimicrobial products used for treat and decontamination inanimate surfaces. In 1998, they first published the Product Performance Test Guidelines, OPPTS 810.2100 Products for Use on Hard Surface – Basic Efficacy Data Requirements used for efficacy testing of disinfectants in collaboration with AOAC International. Lately, it is amended as OSCPP 810.2200: Disinfectants for Use on Hard Surfaces – Efficacy Data Recommendations in September 2012. EPA recommended the carrier tests and use-dilution tests for assessment of disinfectant effectiveness for medical use surface disinfection [81]. Up to date, in cooperation with AOAC International and ASTM International, antimicrobial testing methods & procedures are well documented and specified from EPA’s microbiology laboratory for antimicrobial formulations in the form of liquid, spray and towelette, against *Staphylococcus aureus, Pseudomonas aeruginosa, Salmonella choleraesuis, Mycobacterium bovis* (BCG), *Clostridium difficile*, *Trichophyton mentagrophytes*, non-enveloped viruses (i.e. parvovirus, noroviruses) and *Pseudomonas aeruginosa or Staphylococcus aureus* biofilm. Detailed testing methods are discussed in the following paragraphs.

AOAC Use Dilution Test is a standard operating procedure requested by EPA for evaluating liquid and dilutable liquid disinfectants for hard surfaces. Different series were developed for different microorganism tests - 955.14 (*Salmonella enterica*), 955.15 (*Staphylococcus aureus*), and 964.02 (*Pseudomonas aeruginosa*). The AOAC Use-Dilution Test is a relatively facile method to operate. However, it cannot demonstrate the use of disinfectants in practical conditions. Other methods are also specified by US EPA such as the AOAC METHOD 965.12 Tuberculocidal Activity of Disinfectants, which is a modified version of AOAC Use- Dilution test method applied to justify tuberculocidal efficacy claims for disinfectants. Due to the slow growth rate of the test microorganism (60 days’ incubation time plus an additional 30 days), the test is susceptible to contaminations. AOAC METHOD 955.17 Fungicidal Activity Method, which is designed to access the effectiveness of the disinfectant’s fungicidal activity. In the test, the highest acceptable dilution to disinfect a surface that is contaminated with the fungi in the given contact time is determined. AOAC METHOD 966.04 Sporicidal Activity of Disinfectants is developed to substantiate the sporicidal efficacy of high-level disinfectant or sterilant. By enumerating the number of spores per carrier and the drying the spores on the surface, the test is recognised as a more robust challenge for the rigour of the disinfectant. AOAC METHOD 960.09 Germicidal and Detergent Sanitizing Action of Disinfectants test method is used to validate the efficacy of food contact surface disinfectant/sanitizer with Gram-negative and Gram-positive bacterium. AOAC Germicidal Spray Product Test: 961.02 (Germicidal spray products as Disinfectants) is used to evaluate the efficacy of disinfectant with the spray method on hard, non-porous surfaces. It is a semi-quantitative method based on statistics of passing and far away from real life usage (extreme excess disinfectant quantity per unit surface area). Yet, this method has been modified and used for efficacy assessment of pre-saturated disinfecting towelettes.

ASTM (American Society for Testing and Materials) International is another important standards organization that develops the disinfectant efficacy tests. There are several methods published by ASTM for the effectiveness assessment in terms of different application strategy (liquid, wipes), application area (e.g. food contact surfaces or environmental surfaces and non-porous or porous surfaces etc.) and target microorganism (bacteria, fungi, mycobacteria, spores, biofilm, virus). They are mainly suspension or carrier-based test methods. What highlights in ASTM standards is it embraces several efficacy tests of pre-impregnated towelettes. For instance, ASTM E2362–15, a qualitative method (provide no quantitative reductions) by estimation of growth positive and negative to determine the effectiveness of pre-saturated or impregnated towelettes for hard surface disinfection. The listed materials (apparatus) for testing are easily accessible in a regular microbiology lab. It as well includes a large spectrum of testing organisms including mycobacteria. Similar to ASTM E2362–15 is the ASTM E2896–12, which determining the effectiveness of antimicrobial towelettes with a quantitative Petri plate method instead of a glass slide. Originally designed by Williams et al. in their three-steps protocol to determine the efficacy of disinfectant wipes on surfaces and later amended into standard ASTM E2967–15. In this standard, extra equipment named Wiperator is requested.

In above-mentioned published standards by different world recognised organisations, efficacy tests regarding wipe/towelette are very few and recent. These available quantitative test protocols are critically discussed below.

The EN 16615:2015 is a quantitative test method for the evaluation of bactericidal and yeasticidal activity on non-porous surfaces with mechanical action employing wipes for the medical area. This method displays several advantages allowing the quantitative evaluation of the antimicrobial activity of disinfecting wipes. It is applied to simulate the practical use of disinfecting wipes and allows to detect the cross-contamination caused by the wiping activity. Moreover, it can be used to evaluate the compatibility between the active ingredients in the solution and the wipe materials. It allows a flexible contact time (from 1 to 60 mins), can be tested in both clean and dirty conditions and define the declaration of concentration and exposure time on the disinfectants’ labels. Despite these advantages, this method also displays some drawbacks. The test is considered relatively time consuming, complex and is not possible to strictly control the applied mechanical action. Monotonic test surface (PVC with PUR surface coating) and the test wipe (if not specified by request), as well as the fixed disinfectant volume (16 ml), could significantly influence the outcome. Moreover, it is difficult to discriminate between the microbicidal activity derived from the disinfectant action (which represents the material compatibility issue) and the substrate that could retain microorganism by mere mechanical action.

The Modified AOAC international method 961.02 is meant for the disinfection evaluation performance of pre-saturated towelettes for hard surfaces. It is a simple method to study the variables that could influence the disinfection outcome. Approved by EPA as a method for the registration of spray disinfectants, this method gives a straightforward picture of test products’ performance providing survivor results in the form of a qualitative endpoint (growth positive versus growth negative). However, it exhibits unrealistic results when applied with a large ratio between disinfectant quantity and surface area. Besides, wiping applied pressure cannot be controlled, the concentration of bacteria on the test surface is not standardised, only allows semi-quantitative analysis, can only be applied in a monotonic surface (glass) and it is not possible to evaluate possible cross-contamination. Finally, because it does not address the humidity levels during the drying process of the test surfaces the results can be significantly uncertain.

The ASTM E2896–12 is a quantitative standard test method meant for the determination of the effectiveness of antimicrobial towelettes. The listed materials (apparatus) for testing the wipes are easily accessible in a regular microbiology lab and it requires easy operation procedures to evaluate the disinfecting-wipe ability using glass Petri dishes and corkscrew pattern wiping movements. Simple modifications can be done to test other microbial strain. Also, this method presents some disadvantages such as the lack of control of several variables of the disinfectant-impregnated wipe (disinfectant amount, wipe size, etc.), its exclusive use in monotonic surfaces (e.g. glass Petri dishes), the impossibility to evaluate cross-contamination and the uncontrolled wiping action, especially the wiping pressure. Another important drawback is the inability to differentiating between mechanical removal of inoculum from a surface and chemical inactivation of the test microbe.

The ASTM E2967–15 is a standard test method for assessing the ability of pre-wetted towelettes to remove and transfer bacterial contamination on hard, non-porous environmental surfaces using a specially designed machine to simulate the wiping action, the Wiperator. It allows great precision and reproducibility due to the well-control of the wiping action using the Wiperator. Despite this test is not widely recognised in Europe, it fills some gaps existing in other evaluation methods allowing a realistic contact time and a quantitative evaluation of the antimicrobial activity of disinfecting wipe. Moreover, it guarantees the evaluation of the disinfecting wipe’s ability to remove and prevent the microbial transfer from surfaces and their overall antimicrobial activity. However, also, in this case, the use of a monotonic test surface (stainless steel) and limited contact time (from 5 s up to 45 s) can limit a realistic outcome. There are some critics and debates related to the need for specific extra equipment (Wiperator) and whether the Wiperator could represent a realistic wiping process.

It is clear that an internationally recognized method to guarantees the evaluation of disinfecting wipe’s ability using quasi-realistic conditions, especially regarding test surfaces and cross-contamination, is urgently needed. Standards listed above are able to evaluate the overall antimicrobial efficacy of the testing wipes, but not differentiate the mechanical removal of inoculum from a surface and chemical inactivation of the test organisms. In addition, the wiping strategy should be addressed as one factor that can have an impact on the disinfection efficacy of DIWs. Divergent outcomes with different test standards can be suspected. A guideline for comparable results between various test standards is in demand.

### Efficacy studies in literature

A countable number of studies regarding the efficacy of DIWs have been carried out. Tebbutt et al. may be the first ones, in 1988, to compare the decontamination performance of disposable and reusable disinfectant wipes concluding that the use of disposable disinfectant wipes significantly reduces the risks of cross-contamination. Moreover, their investigation was a breakthrough as it introduced the microbiological assessment of disinfecting wipes efficacy in practical use. The study, not only examined whether the wipe transferred bacteria from one surface to another but also if any organisms remaining on the wipe has been killed [64]. Later, in 1993, Threlkeld et al. compared the disinfecting wipe method with the disinfectant soaking method in their efficacy to eliminate adenovirus 8 from medical instruments. The result revealed that the disinfectant wipe method could readily and thoroughly wipe away the virus from a tonometer and it was more convenient than disinfectant soaking method [40]. However, their finding cannot be safely extrapolated to other equipment items, which implies different target surfaces and organic load that may have an impact on the decontamination performance of disinfecting wipes. In 2007, Williams et al. developed a three-step protocol to quantify the efficacy of disinfectant wipes, their ability to remove and prevent the microbial transfer from surfaces and their overall antimicrobial activity, which could be considered as a milestone for the development of efficacy test for disinfecting wipes [29]. The paper introduced the first stringent test able to assess the ability of antimicrobial wipes to remove, kill and prevent the transfer of bacteria from contaminated surfaces. However, only one wiping material was used in this study, therefore no information can be extrapolated to understand the influence of different wiping materials in the surface disinfection efficacy using wipes. Afterwards, numerable studies have demonstrated the efficacy of disinfecting wipes based on the three-step protocol proposed by William et al. [82]. Siani et al. (2011) tested 9 commercially available wipes from different manufacturers. Their study revealed the importance of application time in the sporicidal activity of disinfecting wipe [82]. However, they did not investigate the role of wiping materials in conjunction with surface disinfectants. One innovation of their study is that they found spore binding to the wipe fibres, which gives more clues about the role of wiping materials in the disinfection process. In addition, the authors introduced the strategy “one wipe, one application, one direction”. Findings from Cadnum et al. gave a clear image of the efficient transfer of *C. difficile* spores from contaminated to clean surfaces using non-sporicidal wipes and the consistent reducing of *C. difficile* spores to undetectable levels at the inoculum site, with no transfer of spores to clean sites, using pre-moistened germicidal wipes [73]. However, the active ingredient of non-sporicidal wipes was not reported.

After several studies with the Williams’ three-step protocol, it has been converted to the ASTM Standard E2967–15. The same year, Sattar et al. (2015) have published a paper regarding the efficacy of bioburden control from surfaces following disinfectant wipes use based on the new ASTM standard E2967–15. Five commercially available wipes have been tested with *Staphylococcus aureus* (ATCC 6538) and *Acinetobacter baumannii* (ATCC 19568) and their performance have been compared [83]. One advance of this research is the newly added drying process, which eliminates the detrimental influence of it on microbial viability. Again, the combination of wiping materials and active ingredients is randomly reported, therefore the study of the interaction issue remains vague. Hernandez et al. (2008) have studied the disinfection performance of chlorine dioxide imbibed wipes against *Mycobacterium avium* based on the European standard prEN 14563 carrier test [25]. However, their study was mainly focusing on mechanical action in the use of disinfecting wipes. There is more than one test method to assess the decontamination efficacy of disinfecting wipes. Gold et al. (2013) in their study have measured the cleanness, bacterial removal, and the force to remove the dried debris. Six commercially available disinfectant wipes were tested [2]. The innovation part of their research is that they also evaluate the force and time required by the disinfectant cleaning wipes to remove the debris from the surface. However, the measurement methods (OPA assay and ATP bioluminescence assay) they adopted seem not to be very accurate. Despite this study gives hints for the selection of the disinfectant cleaning wipes, it is a case study with difficulties to apply for general use. The case study in MRSA-positive hospitalized patients from Cheng et al. (2011) has evaluated the effectiveness of disinfection with wipes against methicillin-resistant *Staphylococcus aureus* (MRSA) [65]. Unfortunately, their experiment design showed a critical drawback. The post-disinfection swab only contained sterile saline solution instead of a neutralizer to counteract the sporicidal action from the disinfectant agent after one prescribed contact time.

The impact of pathogen transfer from fomites to fingers, using surface disinfecting wipes, has been evaluated by Lopez et al. (2014) in their research. Their study tested three different surfaces with four types of microorganisms, *E. coli, S. aureus, B. thuringiensis*, and PV-1. Their study has found that some microorganism may be more resistant to physical removal than others [84], which gives the clue that the adhesion of microorganism on wipes may be different depending on the type of material used.

The impact of the interaction between wiping material and surface disinfectant on the decontamination efficacy of disinfecting wipes was finally taken into account in the work of Engelbrecht et al. (2013). They have tested both cotton and microfiber towels on their abilities to bind quats using three different contact times. The study result indicated the reduction of quats concentration when exposed to cotton fibres, causing the disinfectant to fail the AOAC 961.02 Germicidal spray tests (GSTs) [85]. Unfortunately, the microbiology tests they performed did not test the disinfecting wipe in their field use, because the AOAC 961.02 GST test does not consider the wipe in function of the microorganism removal during the wiping process. Thus, their study proved the deactivation of quats when exposing to cotton towels, but not the decontamination performance of quats disinfecting cotton towels. A list of the most important disinfecting wipes decontamination efficacy tests is summarised in Table 3.

**Table 3:** Disinfecting wipes decontamination efficacy tests in literature

Test organism	Textile substrate	Active ingredient	App. type	Surfaces	Contact time	Test method	Ref.
*E. coli, P. aeruginosa, S. aureus, Streptococcus faecalis*	(a) Heavy-duty paper wipe; (b) Non-woven rayon; (c) Non-woven fabric sheet	(a) 30% ethyl alcohol; (b) 10% ethyl alcohol and cetrimide; (c) Quaternary ammonium compounds	(a,b) PIDW (c) PSDW	Formica boards	Until dry	Swabbing techniques are superior to agar-impression methods	[64]
Adenovirus 8	(1) Pad, (2) Gauze, (3) Pad	(1) 70% isopropyl alcohol, (2) 3% hydrogen peroxide, (3) Iodophor	PSDW	Goldmann tonometer and pneumotonometer tips	5 s for wiping	Quantitatively assayed for residual virus	[40]
*Meticillin-resistant (MRSA) or -susceptible (MSSA) S. aureus*	n.a	Grapefruit extract	PIDW	Stainless steel discs	10 s rotation	Three-step protocol	[29]
*Clostridium difficile*	CAWP	Hypochloride, QACs	PIDW	Steel discs	10 s rotation	Three-stage protocol	[82]
*S. aureus (ATCC 6538)* *Acinetobacter baumannii (ATCC 19568)*	CAWP	H2O2, chloride and chloramine compounds; Sodium hypochlorite 1000 ppm, isopropanol; ethanol, quaternary ammonium compounds	PIDW	Discs (AISI Type 430; 1 cm in diameter and 0.7 mm thick) of magnetized and brushed stainless steel	10 s rotation	ASTM Standard E2967–15	[83]
*Mycobacterium avium*	Ready-to-use wipe	Chlorine dioxide concentration in the activated wipe was 200 ppm.	PIDW	Sterile frosted glass	30 s and 1 min	prEN 14,563	[25]
Coagulated blood test soil, *Streptococcus pneumoniae*	6CAWP	Sodium hypochlorite, hydrogen peroxide, QACs, isopropanol	PIDW	Anesthesia machine surface	n.a	Residual protein debris by o-phthaldialdehyde analysis, bacterial survival by adenosine triphosphate measurement, measure of force required to remove the dried debris	[2]
*MRSA-positive hospitalized patients*	Disposable and nondisposable wipes (100% cotton)	1000 ppm hypochlorite	PIDM PSDM	Bed rails	5 mins	Five-steps method (more information can be found in the article)	[65]
*E. coli, S. aureus, Bacillus thuringiensis spores, poliovirus 1*	CAWP	Quaternary ammonium compounds (QACs)	PIDM	Ceramic tile, laminate, and granite	10 mins	Concentrations of transferred microorganisms on the fingers after the disinfectant wipe intervention	[84]
*S. aureus (ATCC 6538), Salmonella enterica (ATCC 10708), P.aeruginosa (ATCC 15442)*	Cotton and microfibre towels	Quaternary ammonium compounds (QACs)	PSDW	Glass slides	Less than 10 mins	AOAC International method 961.02 Germicidal spray tests (GSTs)	[85]
Campylobacter jejuni	n.a	n.a	PIDW	Ceramic tile, laminate and granite	n.a	Quantitative microbial risk assessment (QMRA)	[86]

Note: *E. coli Escherichia coli, S. aureus Staphylococcus aureus, P. aeruginosa Pseudomonas aeruginosa, CAWP* commercially available wipe product, *PIDM* pre-impregnated disinfecting wipe (pre-wetted disinfecting wipe), *PSDM* pre-soaked disinfecting wipe (bucket method), *n.a.* Not available

## Conclusion and future research

The use of pre-impregnated disinfecting wipes is one of the most efficient and prevalent methods for the decontamination of high-touch environmental surfaces and non-critical medical devices in hospitals and other healthcare centres. There is evidence to support the significance of disinfecting wipes in preventing cross-contamination and spread of HCAIs. Despite this, less is known concerning the effectiveness of disinfecting wipes in the decontamination process. From the studies, several variables influence the disinfection efficacy of DIWs besides the external factors, these include:

- Disinfectant (type, concentration)
- Wipe (material, construction)
- Interaction
- Application method
- Wiping strategy including the applied pressure force, wiped surface area, the geometry of the mechanical action, number of passages, and remaining time on the surface.
- Storage time (function degradation)

Amongst, the interaction between disinfectant and textile substrate is the biggest encumbrance for its disinfection performance. Though, literature has revealed that an inappropriate material of the wipes could interact with the adsorbed active ingredient resulting in lower or even abolished disinfectant efficacy. At present, there is no clear understanding of the interaction phenomenon. Several information gaps have to be filled to obtain consistent and exhaustive knowledge about the interaction, in particular, examining and improving the following issues:

- Material compatibility (combination of wipe and disinfectant)
- Liquor ratio (wipe mass/disinfection solution volume)
- Contact time (of disinfectant and wipes)
- Storage time

Besides, the standards to date remain some drawbacks in testing the effectiveness of DIWs. For example, difficulties exist in differentiating between mechanical removal of inoculum from a surface and chemical inactivation of the test microbe (High risk of cross-contamination when pathogens are just being removed by the wipe instead of being killed by the disinfectant depending on the materials compatibility). More realistic disinfectant volume per unit surface area needs to be improved and applied. Divergent outcomes with different test standards can be suspected. A guideline for comparable results between various test standards is in demand. Nowadays, the most reliable method that can be used in hospitals seems to be the one using ready-to-use disinfecting wipes because of its lower disinfection failure risk. Due to the incomplete study of the decontamination efficacy of DIWs and the lack of testing standards validating the efficacy of DIWs in nosocomial practice, one can hardly advocate for their use in hospitals.

Therefore, one future research direction could focus on the interaction mechanism and its impact on the DIWs’ overall decontamination activity. Furthermore, emphasis should be placed on the ageing of DIWs over storage time in respects to the structure or properties deterioration of the wiping material as well as the antimicrobial efficacy change. Moreover, almost nothing is known about properties, performance and disinfection efficiency of plasma-treated and/or polymer-functionalized wipes. Efficacy test with advanced surface modification technologies can also be considered for future research direction. The development of more environmentally sustained processes is also required considering the waste management of disposable wipes. Additionally, the study on the effectiveness of disinfectant pre-impregnated wipes using the appropriate materials will avoid wasting resources. The outcome research knowledge will be important to ensure hospitals daily workflow from unnecessary risk of infection outbreak and to complement the products’ user manual of disinfectant and wipes in the market.

## Data Availability

Not applicable.

## Abbreviations

ADBAC: Alkyl-dimethyl-benzyl-ammonium chloride
AHP: Accelerated hydrogen peroxide
AOAC: Association of Official Analytical Chemists
ASTM: American Society for Testing Materials
CAWP: Commercially available wipe product
CDC: American Centres for Disease Control and Prevention
CEN: European Committee for Normalization
DIW: Disinfectant-impregnated wipe
ECHA: European Chemicals Agency
EPA: Environmental Protection Agency
GSTs: Germicidal spray tests
HCAIs: Healthcare-associated infections
HICPAC: Healthcare Infection Control Practices Advisory Committee
MRSA: Methicillin-resistant *Staphylococcus aureus*
NCBI: National Center for Biotechnology Information
OPP: Office of Pesticide Programs
OPPTS: Office of Prevention, Pesticides & Toxic Substances
PAA: Peracetic acid
PIDM: Pre-impregnated disinfecting wipe (pre-wetted disinfecting wipe)
PSDM: Pre-soaked disinfecting wipe (bucket method)
QACs: Quaternary Ammonium Compounds (quats)
QMRA: Quantitative microbial risk assessment
RTUDW: “Ready-to-use” disinfecting wipe
TC 216: Technical Committee 216
